# Technetium Immobilization on Carbon Steel Corrosion Products Under Simulated Geological Radioactive Waste Repository Conditions

**DOI:** 10.3390/ma18225220

**Published:** 2025-11-18

**Authors:** Elena Abramova, Grigoriy Artemiev, Konstantin German, Alexey Safonov

**Affiliations:** Frumkin Institute of Physical Chemistry and Electrochemistry (IPCE), Russian Academy of Sciences (RAS), 31-4, Leninsky Prospect, Moscow 119071, Russia; gorchicta246@mail.ru (E.A.); artemyev56@gmail.com (G.A.); guerman_k@mail.ru (K.G.)

**Keywords:** carbon steel, corrosion products, technetium-99, ferrihydrite, reduction, geological radioactive waste repository

## Abstract

The migration of the long-lived isotope technetium-99 (half-life 2.1 × 10^5^ years) presents a significant challenge for the deep geological disposal of radioactive waste. This study investigates the immobilization of technetium by carbon steel corrosion products under aerobic and anaerobic conditions simulating the Yeniseysky site (Krasnoyarsk Region, Russia), a proposed location for a Deep Geological Repository (DGR). Over time, the degradation of barrier materials is expected to allow low-salinity solutions to be brought into contact St3 steel, the intended container material for vitrified radioactive waste in the Russian context, leading to crevice corrosion. The findings demonstrate that carbon steel containers act not merely as a physical barrier but also as a chemical barrier by facilitating the reductive immobilization of technetium. The most effective reduction of technetium was observed in the presence of ferrihydrite as a corrosion product under both aerobic and anaerobic conditions, as indicated by distribution coefficient (*K_d_*) values ranging from 1.4 × 10^3^ to 1.6 × 10^3^ cm^3^/g. However, the presence of bentonite clay can diminish the efficiency of this process by adsorbing corrosion products, resulting in a 50% reduction in the distribution coefficients. In contrast, leaching products from aluminophosphate glass and cement had a less pronounced effect on technetium immobilization, causing a decrease in distribution coefficients of no more than 30%. The results of this research can be applied to model the long-term behavior of technetium in the evolving environment of a geological radioactive waste repository.

## 1. Introduction

The environmental migration of the long-lived isotope technetium-99 (half-life 211,000 years) presents a significant challenge in the contexts of radiation accidents, nuclear testing, and the disposal of radioactive waste [1,2]. This challenge primarily stems from the high solubility and environmental mobility of its oxidized form, the pertechnetate anion (TcO_4_^−^) [3,4]. Consequently, its potential for accumulation in humans and animals poses a considerable radiobiological risk [2].

Modern radioactive waste management strategies involve its disposal in deep geological formations in solidified states, employing a multi-barrier safety system. However, given technetium’s long half-life and high migratory potential, waste containing this radionuclide requires particular caution and enhanced containment measures [5,6,7].

A prevalent method for technetium immobilization is its reductive precipitation into the sparingly soluble form of Tc(IV) [8,9,10,11]. Among various reducing agents, iron compounds, such as zero-valent iron (ZVI) and slags, are most frequently employed for the reduction of pertechnetate [12,13]. This principle has been applied in several experimental studies for the in situ immobilization of technetium via the introduction of ZVI [14]. Furthermore, during the formation of iron (oxy)hydroxide films, technetium can, in some cases, be incorporated into the developing crystal lattice, providing an additional mechanism for long-term retention [15,16].

Carbon steel is a commonly employed material for the construction of containers in Deep Geological Repositories (DGRs) for radioactive waste [17,18]. Over the long-term evolution of a DGR, the corrosion of these steel containers is anticipated to be a critical factor influencing the environmental behavior and mobility of sequestered technetium.

The corrosion of carbon steel in an oxidizing environment is an electrochemical process involving the coupled anodic dissolution of iron (Fe → Fe^2+^ + 2e^−^) and the cathodic reduction of oxygen (O_2_ + 2H_2_O + 4e^−^ → 4OH^−^). This process generates a succession of iron mineral phases. Initially formed iron(II) (oxy)hydroxides are subsequently oxidized to more stable iron(III) compounds in the presence of oxygen and water, leading to the progressive degradation of the material.

Oxygen consumption due to aerobic corrosion can lead to anoxic conditions. The time required to achieve anaerobic, anoxic conditions will depend on the rate and manner in which the repository becomes water-saturated [19]. Under anoxic conditions, steel will continue to corrode via direct reaction with water through the following reaction (anaerobic corrosion) [20,21]: Fe + 2H_2_O → Fe(OH)_2_ + H_2_. Since Fe(OH)_2_ is thermodynamically unstable, it can be converted to magnetite in a second reaction step, known as the Schikorr reaction [21]: 3Fe(OH)_2_ → Fe_3_O_4_ + 2H_2_O + H_2_. Thus, the overall reaction is: 3Fe + 4H_2_O → Fe_3_O_4_ + 4H_2_. Although Fe_3_O_4_ is the thermodynamically preferred end product, Fe(OH)_2_ may actually dominate at temperatures below 60 °C, as the Schikorr reaction can be kinetically hindered [22,23].

Research into technetium retention under such aerobic conditions has demonstrated its sorption to iron corrosion products like wüstite, magnetite, and hematite [24]. The system, governed by oxidizing conditions, precludes the formation of strong complexes. Within this environment, Fe^2+^ ions facilitate the reductive immobilization of Tc(VII), as pertechnetate (TcO_4_^−^), to the less soluble Tc(IV) state, a process concomitant with the formation of ferric hydroxide (Fe(OH)_3_). This transformation can be represented by the reaction: 3Fe^2+^ + TcO_4_^−^ + (n + 7)H_2_O ↔ 3Fe(OH)_3_ + TcO_2_·nH_2_O + 5H^+^.

Furthermore, the precipitated Tc(IV) may form aggregates and deposit onto the surfaces of iron minerals, potentially as monomeric or dimeric TcO_2_ complexes [25], providing an additional mechanism for long-term sequestration. Consequently, evaluating the influence of steel corrosion products on technetium immobilization is critical for predicting radionuclide behavior within the engineered barrier system and the surrounding geosphere of a disposal facility. Such an assessment requires a multifactorial approach, integrating site-specific geochemical data and accounting for the presence of other engineered barrier materials.

This study focuses on the Yenisei site in Krasnoyarsk Krai, a candidate location for a Deep Geological Repository (DGR). The investigation employed carbon steel grade St3—a material analogous to A284Gr.D and A570(36) (USA) and considered for radioactive waste packaging [26,27]—along with cement and bentonite clay as representative safety barriers [28,29]. The primary objective of this work was to quantify the impact of corrosion processes on technetium mobility. This was investigated using the corrosion-susceptible carbon steel under conditions simulating the DGR environment, with specific attention to the effects of coexisting materials, namely the aluminophosphate glass waste form, bentonite clay, and cement.

## 2. Materials and Methods

### 2.1. Materials

This study utilized carbon steel grade St3 specimens with a nominal composition (wt.%) of 0.12% C, 0.8% Si, 0.5% Mn, 0.04% P, 0.04% S, 0.1% Cr, 0.3% Ni, 0.1% Cu, and balanced Fe. The specimens were fabricated as rectangular plates measuring 15 × 10 × 1.5 mm. St3 is a Russian standard carbon steel of ordinary quality, regulated under GOST (Russian State Standard) 380-2005 [30]. Prior to experimentation, all samples were subjected to a 25-min ultrasonic cleaning process in a Sapphire-0.8 TC ultrasonic bath (Smirdex, Moscow, Russia) using a 1:1 (*v*/*v*) mixture of ethanol (C_2_H_5_OH) and toluene (C_7_H_8_) to remove surface contaminants. The aqueous phase consisted of a synthetic model solution with a total ionic strength of 10^−3^ M, designed to simulate the groundwater chemistry of the Yenisei site, a proposed location for a future Russian DGR [26,31]. The solution had the following composition: Mg^2+^—4.95, Ca^2+^—12.2, K^+^—1.15, SO_4_^2−^—4.95, Cl^−^—25.6, HCO_3_^−^—8.87 (all values in mg/L), and an initial pH of 7.1.

### 2.2. Experimental Setup

#### 2.2.1. Experiments with Carbon Steel Plates

Carbon steel plates were immersed in 100 mL glass vials containing the model solution at a solid-to-liquid (S:L) ratio of 1:20 (Sample MW-plate). The solution was spiked with sodium pertechnetate (Na^99^TcO_4_, JSC Isotop, Moscow, Russia) to achieve a final volumetric activity of 300 Bq/mL. Liquid phase sampling was conducted over a period of one week, until the solution activity reached an equilibrium state. The control systems were: model water with pertechnetate ions (without steel), as well as steel samples in model water with added TcO_4_^−^ and the IFKhAN-29 inhibitor (Frumkin Institute of Physical Chemistry and Electrochemistry (IPCE), Russian Academy of Sciences (RAS), 31-4, Leninsky Prospect, 119071 Moscow, Russia) [32], under both aerobic and anaerobic conditions.

The evolution of the corrosion layer was analyzed using scanning electron microscopy (SEM) and X-ray photoelectron spectroscopy (XPS). These analyses were performed on dried steel samples retrieved after two key exposure intervals: 24 h, corresponding to the period of peak corrosion formation, and 7 months, marking the experiment’s endpoint.

#### 2.2.2. Sorption Experiments with Synthesized and Commercial Iron Phases

To isolate corrosion products for dedicated sorption tests, St3 plates were corroded in the model solution for 7 days. The resulting solids were then collected via filtration and air-dried at ambient temperature.

In parallel, experiments were conducted using commercially available and synthesized iron minerals to simulate specific corrosion phases. These included magnetite (Fe_3_O_4_) and FeO (CAS No.: 1400866 Sigma-Aldrich, St. Louis, MO, USA), alongside goethite (FeO(OH)) and hematite (Fe_2_O_3_) (NPO “EcoTek”, St. Petersburg, Russia). Freshly precipitated ferrihydrite (Fe^3+^_10_O_14_(OH)_2_) was synthesized according to the procedure outlined in [33]; its phase composition, along with that of the other minerals, is provided in the Supplementary Material, Figure S1.

For all sorption experiments, the solid phase (either the harvested corrosion products or a synthetic iron mineral) was brought into contact with the simulated groundwater (S:L = 1:20). The initial ^99^Tc activity, introduced as pertechnetate (TcO_4_^−^), was 300 Bq/mL. The study was performed under aerobic and anaerobic regimes. Anaerobic conditions were achieved by evacuating the gas phase followed by purging with extra pure argon. The distribution coefficient (*K_d_*) was determined at the 1-month interval, under equilibrium conditions. Samples were collected at 1 h, 1 day, and subsequently at weekly intervals. The systems were monitored for one month, with periodic sampling until solution activity stabilized, indicating sorption equilibrium. The aqueous concentration of pertechnetate was quantified throughout using scintillation counting.

#### 2.2.3. The Effect of Barrier Components on the Immobilization of Technetium

To evaluate the influence of other engineered barrier materials, sorption experiments were conducted with corrosion products in the presence of leachates from Portland cement (CEM I) and aluminophosphate glass, as well as in a suspension of bentonite clay.

Sample MWC: Corrosion products were brought into contact with a 1:1 mixture of model water and CEM I cement leachate.

Sample MWG: Corrosion products were brought into contact with a 1:1 mixture of model water and aluminophosphate glass leachate.

Sample MWB: Corrosion products were brought into contact with a suspension of “10th Khutor” bentonite clay (S:L = 1:10). The composition of the clay is provided in [34].

Preparation of Barrier Material Leachates:

CEM I Cement Leachate: Cubic cement matrices (2 × 2 × 2 cm) were prepared with a water-to-cement ratio of 0.5. After 28 days the matrices were immersed in simulated water for an additional 28 days to generate the leachate. The resulting cement-leached water, used in subsequent experiments, had the following representative composition (mg/L): K—141.9, Si—23.7, Ca—1284, Na—281, SO_4_^2−^—112, HCO_3_^−^—78, pH 11.5.

Aluminophosphate Glass Leachate: The leachate was prepared using glass with a composition (in wt.%) of Na_2_O—20.63, Al_2_O_3_—23.62, and P_2_O_5_—45.72, as previously described [35]. The glass was ground in a corundum mortar to a particle size of <0.05 mm. A static leaching test was performed by bringing 1 g of this glass powder into contact with 19 mL of model water (S:L = 1:20) in a polypropylene vial for 28 days. The final leachate composition for macrocomponents was (mg/L): K—7, Si—5.7, Ca—18.8, Al—0.06, Fe—0.37, Na—116.9, HPO_4_^2−^—30, PO_4_^3−^—16, pH 9.9.

### 2.3. Analytical Techniques

The surface area affected by corrosion was quantified through optical microscopy. Analysis was performed using a G1000 microscope (Srate Optical Instrument Manufactory, Nanyang, China) at 2× magnification, coupled with a CamRec Expert-009 2D camera system (Zhejiang Uniview Technologies Co., Ltd., Hangzhou, China). The acquired images, with a resolution of 2048 × 1536 pixels and covering an observed area of 2 cm^2^, were processed and analyzed using ImageJ software 1.54p to determine the lesion area.

The activity of technetium-99 in the aqueous phase was measured with a PerkinElmer Tri-Carb 3180 TR/SL liquid scintillation analyzer (PerkinElmer, Shelton, CN, USA).

The efficiency of technetium immobilization onto the solid phases was evaluated by calculating the interphase partition coefficient (*K_d_*, in cm^3^/g) according to Equation (1):

(1) $$K_{d}=\left(\frac{A_{0}-A}{A}\cdot \frac{V}{m}\right)$$

where A_0_ and A are the initial and current volumetric activities of the solution (Bq/mL), respectively; *V* is the volume of the liquid phase (mL); and *m* is the mass of the solid phase (g).

Desorption of technetium was carried out using the Tessier method [36] modified for technetium [37,38].

The surface morphology and elemental composition of the samples were analyzed by a Tescan MIRA3 scanning electron microscope (SEM) (TESCAN, Brno, Czech Republic) with an Oxford Ultim Max 100 EDS analyser (Oxford Instruments, High Wycombe, UK). Prior to analysis, the samples were extracted from the liquid medium, dried at room temperature, and mounted on aluminum stubs with conductive tape. To enhance conductivity, the samples were coated with a carbon layer using a Q150T E Plus sputter coater (Quorum Technologies, West Sussex, UK) under a vacuum of 4 × 10^−3^ Pa and a current of 50 A. SEM imaging was performed using both secondary electron (SE) and backscattered electron (BSE) modes at an accelerating voltage of 20 kV.

The surface composition was characterized by X-ray photoelectron spectroscopy (XPS) on an OMICRON ESCA+ system (Scienta Omicron, Uppsala, Sweden). The spectrometer was operated with an aluminum non-monochromatic anode (Al Kα = 1486.6 eV) at a power of 252 W (DAR 4000, Scienta Omicron). Using an Argus analyzer-detector (intec Gesellschaft für Informationstechnik mbH, Lüdenscheid, Germany), survey and high-resolution spectra were recorded at a constant pass energy of 20 eV. The C 1s peak at 285.0 eV served as an internal standard for charge correction. The analysis chamber maintained a base pressure of <10^−9^ mbar during data collection, and the spectral background was corrected using the Shirley algorithm [39]. The spectral fitting procedure was carried out using the CasaXPS 2.3.25 software.

X-ray diffraction data were collected on a Panalytical Aeris compact X-Ray diffractometer (Malvern Panalytical, Malvern, UK) with a cobalt-anode X-ray tube (wavelength Kα (Cu Kα) = 1.54184 Å), V = 40 kV, I = 15 mA, step size 0.005°2θ, acquisition time of 30.6 s/step and measurement range 10–65°2θ. X-ray diffraction data were interpreted using HighScorePlus software v5.3a with PDF2 database.

## 3. Results

### 3.1. Interaction of Pertechnetate Ions with Carbon Steel Samples During Their Corrosion

**Figure 1:**
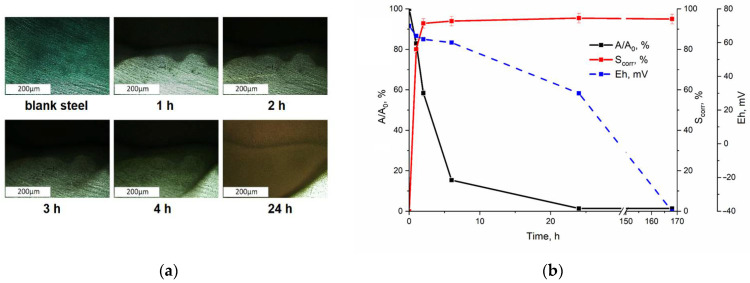
The efficiency of Tc removal over time in the model water of the DGR upon contact with a plate of St3 steel: (**a**) visual change in the steel surface recorded using optical “in situ” microscopy in the model solution, (**b**) kinetics of the decrease in Tc activity with St3 steel in the model water.

Visual analysis of the St3 carbon steel plate surface (Figure 1a) indicated that corrosion initiation occurred almost immediately upon immersion in the solution. The corrosion front progressed rapidly, covering approximately 50% of the surface area within 6 h and exceeding 95% after 24 h.

Concurrent with the corrosion development, a significant decrease in the aqueous-phase technetium concentration was observed (Figure 1b). Within the first 6 h, the ^99^Tc activity (A/A_0_) dropped to 20% of its initial value, achieving a removal efficiency of 98% after 24 h.

Notably, this rapid technetium immobilization took place under oxidizing conditions, as evidenced by redox potential (Eh) values ranging from +80 to +60 mV. A transition to reducing conditions (Eh < 0 mV) was not observed until the fifth day of the experiment, stabilizing at a final value of −40 mV for the remaining duration. A control experiment conducted in the absence of a steel plate showed no measurable technetium removal, confirming the critical role of the steel corrosion process (see Supplementary Table S1). The removal of technetium from a solution with steel in the presence of a corrosion inhibitor did not exceed 1.4% by the end of the experiment under both aerobic and anaerobic conditions.

**Figure 2:**
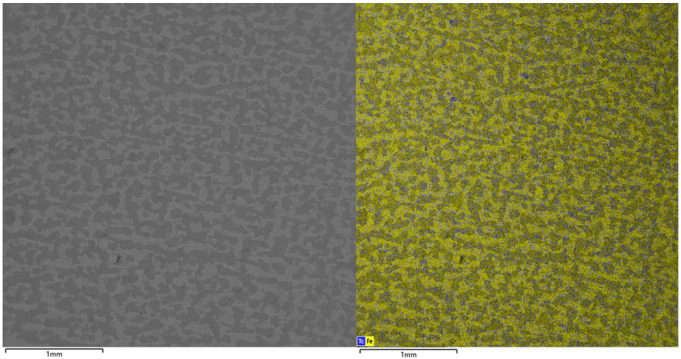
Distribution maps of Fe (yellow) and Tc (blue) on the steel surface after 24 h of contact.

EDS mapping revealed the spatial distribution of technetium and iron across the corroded steel surface. The analysis revealed a strong correlation between the technetium accumulation and the distribution of iron-based corrosion products across the substrate (Figure 2). Furthermore, discrete zones of elevated technetium concentration were identified, indicating localized enrichment of the radionuclide.

### 3.2. Characterization of Corrosion Products

The corrosion products on the steel plate were characterized after 1 day and 1 week of immersion using scanning electron microscopy with energy-dispersive X-ray spectroscopy (SEM-EDS), complemented by X-ray fluorescence (XRF) spectroscopy.

#### 3.2.1. Electron Microscopy Analysis

It is important to note that EDS analysis is limited in its ability to detect light elements, such as hydrogen. Consequently, the identification of iron-bearing mineral phases was based on the presence of Fe and O in the composition and the morphology of the analyzed points.

**Figure 3:**
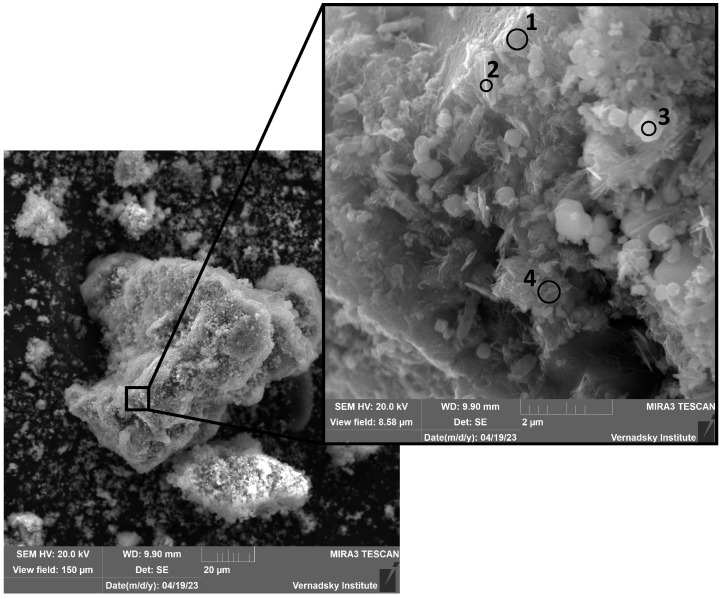
Micrographs of corrosion products in model water after 24 h of the experiment. The elemental composition of the corrosion products in the zones marked in Figure 3 is given in the Table S2.

**Figure 4:**
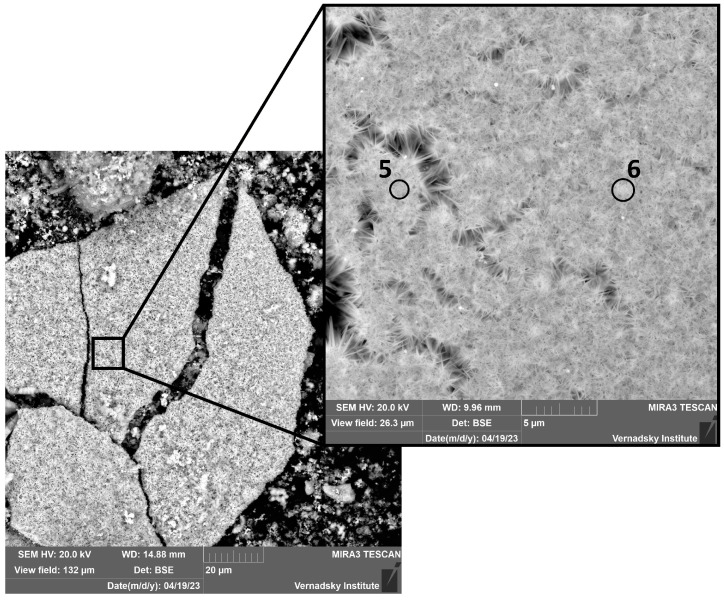
Detailed view of corrosion products in the form of goethite crystals after 24 h of the experiment. The elemental composition of the corrosion products in the zones marked in Figure 4 is given in the Table S2.

One day after immersion (Figure 3), several distinct phases were identified. A porous, layered matrix, consistent with the morphology of ferrihydrite (point 1) [40], served as a substrate for needle-shaped aggregates, tentatively identified as goethite (points 2, 4) [41]. Additionally, cubic and spherulitic crystals, potentially corresponding to magnetite or hematite (point 3) [42,43,44,45], were observed. Isolated plate-like aggregates composed exclusively of acicular goethite crystals (points 5, 6) were also identified (Figure 4).

The elemental composition of the corrosion products in the zones marked in Figure 3 and Figure 4 is given in the Supplementary Materials, Table S2.

**Figure 5:**
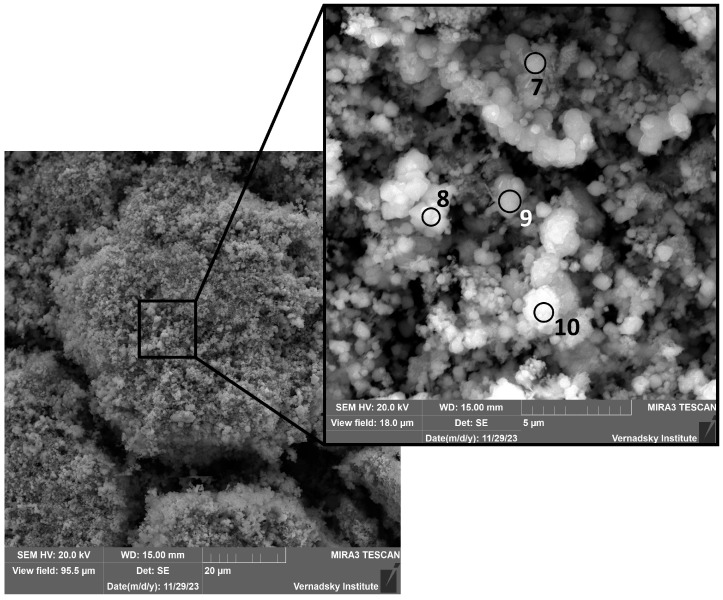
Micrographs of corrosion products in model water after 1 week. The elemental composition of the corrosion products in the zones marked in Figure 5 is given in the Table S2.

Figure 5 shows micrographs obtained one week after steel making contact with the solution. They reveal mineral assemblages including well-defined octahedral and cubic magnetite crystals, as well as spherolitic formations that could be represented by either magnetite or hematite (α-Fe_2_O_3_). The corrosion products have a distinct crystal structure and vary in size. Analysis revealed the almost complete absence of goethite and ferrihydrite, indicating the formation and development of more stable mineral phases.

#### 3.2.2. Identification of Corrosion Products by XPS

**Figure 6:**
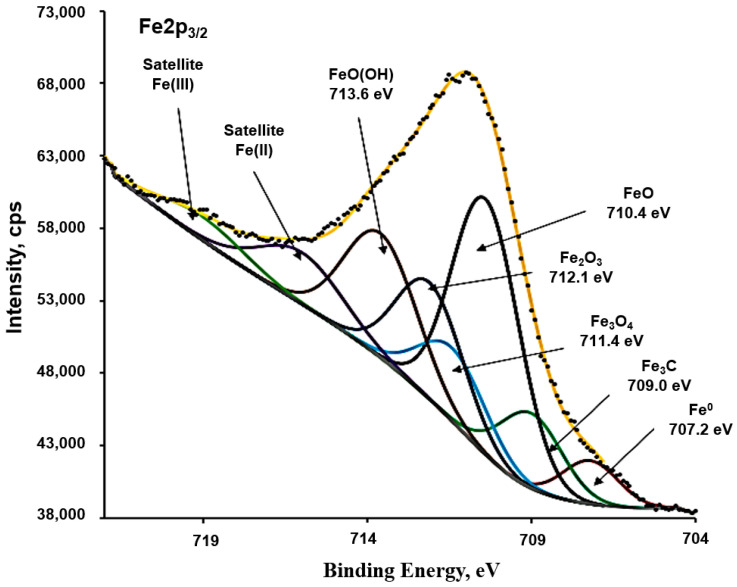
XPS spectrum of the surface of carbon steel with corrosion products.

XPS analysis (Figure 6) confirmed the complex nature of the corrosion layer. A detailed analysis of the electronic states was performed by employing a curve-fitting procedure adapted from reference [46]. The high-resolution Fe 2p_3_/_2_ spectrum was fitted with eight individual components, representing the different chemical states of iron. The identified species were: Fe^0^ (707.2 eV), iron carbide (709 eV), FeO (710.4 eV), Fe_3_O_4_ (711.4 eV), Fe_2_O_3_ (712.1 eV), and FeO(OH) (713.6 eV). The fit also accounted for the characteristic satellite structures of Fe(II) and Fe(III), which are essential for accurate interpretation of iron spectra. This diversity of phases suggests a multi-stage corrosion process under the given conditions.

### 3.3. Evaluation of the Oxidation State of Technetium on a Steel Surface

**Figure 7:**
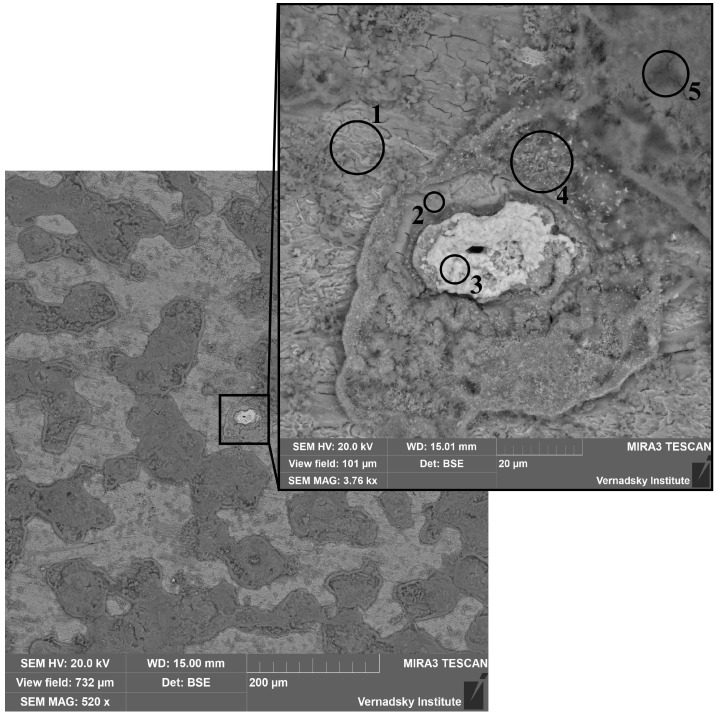
Tc inclusions on a steel plate after 24 h.

The presence of iron–technetium phases was confirmed by SEM-EDS, directly correlating with regions of elevated technetium content. The EDS data suggest a mechanism of coprecipitation, whereby Tc(IV) may be incorporated into the structure of the iron corrosion products. As shown in Figure 7, the formation surrounding the phase indicated by point 3 lacks a well-defined crystalline structure (point 2) and exhibits a morphology consistent with ferrihydrite, which is in agreement with observations from previous micrographs. Surrounding this formation (points 4 and 5, upper right corner and the lower part of the enlarged image), aggregates are evident. These aggregates are predominantly composed of acicular crystals, the morphology of which is similar to the goethite phases identified earlier (as Figure 4). Furthermore, the presence of brighter particles at point 4 indicates that the steel corrosion process involves not only the reduction of technetium, resulting in the formation of an iron-Tc(IV)-containing phase (point 2), but also the sorption of technetium onto the goethite matrix. In the upper left corner and the upper section of the image, cubic and spherical crystals are observable; these are tentatively identified as either magnetite, hematite, or a mixture of these phases. The feature marked as point 1, along with similar structures, is identified as the surface of the steel plate.

The elemental composition of the corrosion products in the zones marked in Figure 7 is given in Table 1.

**Figure 8:**
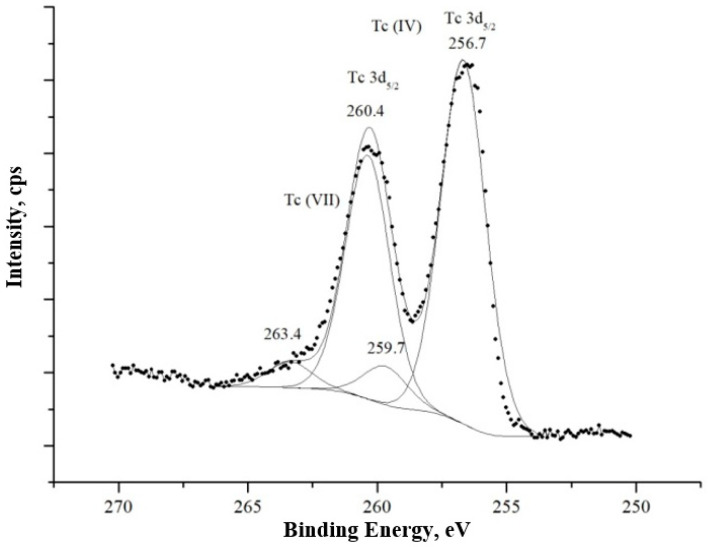
XPS spectrum of Tc on the surface of the plate after 24 h.

The chemical state of technetium was investigated using X-ray photoelectron spectroscopy (XPS). A comprehensive description of this software has been provided in reference [47]. As evidenced by the data in Figure 8, the predominant species was identified as Tc(IV). This conclusion is primarily based on the presence of a distinct peak at a binding energy of 256.7 eV, which is a definitive signature for the +4 oxidation state. The line shape and the positions of the principal peaks in the acquired spectrum correspond accurately with the comprehensive spectral interpretation provided in our earlier study [48].

### 3.4. Assessing the Role of Corrosion Products in Technetium Immobilization

The corrosion of the steel sample yielded a succession of iron (oxy)hydroxide phases. Initial, metastable phases such as ferrihydrite formed under oxidizing conditions, with subsequent transformation into more stable crystalline phases, notably magnetite and hematite.

To quantitatively evaluate the role of individual phases in technetium sequestration, model sorption experiments were conducted under both aerobic and anaerobic conditions using both a harvested mixture of corrosion products and pure mineral standards (Table 2).

Under aerobic conditions, the mixture of separated corrosion products demonstrated high efficiency, removing 96.6% of technetium from the solution. Analysis of the pure phases revealed significant differences in their sorptive capacities. The highest distribution coefficients (*K_d_*) were observed for FeO and ferrihydrite, with values of 1798 and 1646 cm^3^/g, respectively. In stark contrast, magnetite, goethite, and hematite exhibited markedly lower affinities for technetium, with *K_d_* values ranging from only 0.6 to 1.1 cm^3^/g.

Under anaerobic conditions, a significant decrease in both the technetium removal efficiency and the distribution coefficient (*K_d_*) was observed for the harvested mixture of corrosion products, with values of 34.2% and 134.8 cm^3^/g, respectively. This stands in contrast to their performance under aerobic conditions.

Conversely, the sorption parameters for the well-crystallized phases—magnetite, goethite, and hematite—remained consistently low and were largely independent of the redox regime.

### 3.5. Analysis of Technetium Forms on Corrosion Products by the Stepwise Desorption Method

**Figure 9:**
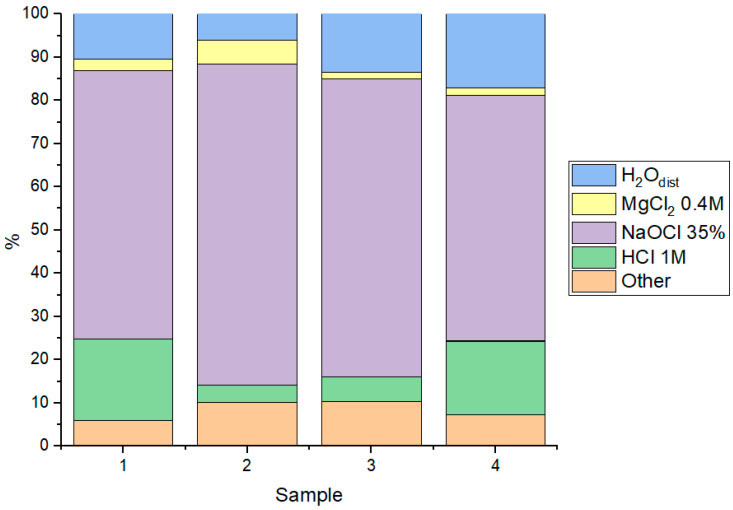
Desorption by the Tessier method (1—corrosion product powder; 2—plate with corrosion products; 3—ferrihydrite; 4—FeO).

Sequential chemical extraction of the technetium-sorbed solids, performed according to the Tessier method, demonstrated that for all investigated phases, the predominant fraction of technetium was released in the step targeting reducible species (NaOCl 35%). This confirms that the immobilized technetium was predominantly in its reduced form (Figure 9).

The combined contribution of the readily exchangeable and weakly adsorbed fractions (extracted by distilled water and magnesium chloride) did not exceed 15% for any material, indicating a strong and largely irreversible fixation of technetium to the solid matrices. As anticipated, the ionic (exchangeable) form of technetium played a negligible role in its overall immobilization.

The fraction of technetium associated with the solid phase and released by 1 M HCl extraction—representing forms more strongly bound to the corrosion products—accounted for 10–15% in the case of the bulk corrosion product powder and FeO. For ferrihydrite, this fraction was less than 5%. Notably, a significant portion (5–10%, varying by material) was identified in the residual, firmly fixed form, suggesting potential incorporation of technetium into the mineral lattice structure.

### 3.6. The Influence of Engineering Safety Barrier Components on the Immobilization of Tc on Corrosion Products

The kinetics of technetium immobilization by corrosion product powder in the presence of various engineered barrier components are summarized in Table 3, which presents the temporal evolution of the relative technetium concentration (A/A_0_) and the corresponding distribution coefficients (*K_d_*). For all samples, including the baseline condition without additives, the primary sorption process occurred within the first 24 h, with the system reaching a stable sorption equilibrium that persisted until the 168-h endpoint. Comparative analysis revealed that the presence of bentonite clay (Sample MWB) resulted in a lower overall technetium sorption efficiency compared to the baseline scenario.

The influence of engineered barrier components on technetium immobilization is quantitatively reflected in the distribution coefficient (*K_d_*) values. The *K_d_* values measured in the presence of bentonite clay (MWB) were approximately four times lower than those observed in the additive-free control. In contrast, the presence of aluminophosphate glass and cement leachates resulted in a more moderate reduction of approximately 20% in the *K_d_* values.

## 4. Discussion

Immersion of the St3 steel plate in simulated groundwater, representing the physicochemical conditions of the Yenisei site, resulted in areal corrosion that progressed to over 95% of the surface within 24 h under initial aerobic conditions. Analysis of the corrosion products identified a mineral assemblage characteristic of such an environment, including ferrihydrite, goethite, magnetite, and hematite.

The phase composition evolved in response to the shifting redox conditions. During the initial aerobic stage, metastable phases such as green rust—layered double hydroxides containing both Fe^2+^ and Fe^3+^ ions [49]—were formed. The oxyhydroxide mixture was predominantly composed of ferrihydrite (Fe^3+^_10_O_14_(OH)_2_), a metastable, poorly crystalline phase that acts as a precursor to more thermodynamically stable minerals [50]. As oxidizing conditions persisted, ferrihydrite underwent dehydration and recrystallization, forming more stable, crystalline phases, primarily goethite and hematite. Corrosion products examined after 24 h exhibited a range of morphologies, from the amorphous aggregates characteristic of ferrihydrite to cryptocrystalline clusters and well-defined acicular goethite crystals.

A transition to anaerobic conditions by the fifth day of the experiment led to the formation of magnetite (Fe_3_O_4_) as the predominant phase [51]. This observation is consistent with thermodynamic modeling of St3 steel anaerobic oxidation, which identifies magnetite as the primary corrosion product [52]. While other models, such as that in [53], calculate Fe(II) hydroxide and siderite, magnetite or Fe(III) hydroxide for anaerobic scenarios, the model in [52] specifically accounts for the chemical kinetics of oxidation, solid-phase formation, and surface passivation by corrosion products. Thus, at the end of the experiment, the corrosion products comprised not only Fe^3+^ compounds but also insoluble iron species of mixed valence (Fe^2+^/Fe^3+^). In the mildly reducing environment (−40 mV), complex mineral associations formed, predominantly consisting of magnetite; however, the complete reduction of Fe^3+^ compounds was not observed. Furthermore, driven by the system’s tendency to form more stable phases, the formation of hematite was identified [54]. The corrosion rate calculated for the initial 24-h period in this study was 5.8 μm/year. In our experiment (Section 3.1), the mass loss of the iron plate was 0.47 mg. This mass loss corresponds to the formation of 0.6 mg of corrosion products (calculated as FeO), equivalent to 8.3 × 10^−6^ mol. The liquid phase contained 0.024 mg of Tc, or 2.4 × 10^−7^ mol (based on an initial volumetric activity of 300 Bq/mL and a solid-to-liquid ratio of 1:20). Based on these data, the calculated ratio of pertechnetate to corrosion products in the experiment was 1:35. This ratio resulted in the rapid and quantitative reduction of pertechnetate within the first few hours of the experiment. Thus, technetium immobilization was observed to occur during the initial oxidative phase of corrosion, with reductive precipitation identified as the primary mechanism. Within 24 h, this process achieved a pertechnetate removal efficiency exceeding 98% from the aqueous phase.

XPS analysis consistently confirmed the reduction of Tc(VII) to Tc(IV), with technetium predominantly identified as TcO_2_ in the corrosion product sediment. Furthermore, scanning electron microscopy revealed the presence of technetium dioxide phases with dimensions up to 10 microns in samples analyzed after 7 days. This finding was corroborated by sequential chemical extraction, which indicated that the majority of the sequestered technetium existed in a reduced, non-exchangeable form.

The reduction of pertechnetate is facilitated by the development of localized reducing microenvironments and the presence of suitable reductants [55]. In this system, Fe^2+^ ions, released during the early stages of steel corrosion, likely serve as the primary electron donor. Concurrently, the rapid consumption of dissolved oxygen during the oxidation of metallic iron (Fe^0^) promotes the formation of anoxic zones at the steel surface. Within these localized environments, the reductive transformation of Fe(III) oxyhydroxides can lead to the formation of more stable, crystalline phases containing Fe(II) or mixed-valence iron species. A representative mineral of this latter category is magnetite (Fe_3_O_4_).

A schematic representation of the proposed reaction pathways is provided below.

(1) Fe^0^ Oxidation

(A) Fe^0^ − 1e^−^ → Fe^2+^*/*(K) O_2_ + 2H_2_O + 4e^−^ → 4OH^−^ (2)

(2) Fe^2+^ Oxidation

(A) Fe^2+^ − 1e^−^ → Fe^3+^*/*(K) 2H_2_O + 2e^−^ → H_2_↑ + 2OH^−^ (3)

(3) Oxidation of iron (II) to magnetite under anaerobic conditions (the resulting Fe^3+^ ions and the remaining Fe^2+^ co-precipitate to form magnetite Fe_3_O_4_)

Fe^2+^ + 2Fe^3+^ + 8OH^−^ → Fe_3_O_4_ + 4H_2_O (4)

(4) Pertechnetate Reduction by ion Fe^2+^

3Fe^2+^ + TcO_4_^−^ + (n + 7)H_2_O ↔ 3Fe(OH)_3_ + TcO_2_·nH_2_O + 5H^+^ (5)

Evaluation of the role of individual model corrosion products in technetium removal indicates that the process occurs most effectively under oxidizing conditions, with ferrihydrite and FeO serving as the primary phases responsible for sequestration. This is substantiated by the significantly lower distribution coefficients (*K_d_*) measured under anoxic conditions compared to oxic ones, suggesting that surface-mediated reduction is a dominant mechanism.

In contrast, magnetite, goethite, and hematite exhibited a minimal capacity for pertechnetate uptake, regardless of the redox regime. The limited reactivity of goethite and hematite can be attributed to the exclusive presence of Fe(III) in their structures, which is incapable of acting as a reductant. Although magnetite contains structural Fe(II), it was not reactive towards technetium reduction under the experimental conditions considered [9], likely due to its stable spinel structure limiting electron transfer.

The potential incorporation of technetium into the crystalline lattice of iron oxides, as suggested in several studies, is indirectly supported by the identification of a non-exchangeable fraction in the sequential extraction data. For the bulk corrosion products and ferrihydrite, this recalcitrant fraction constituted approximately 10% of the immobilized technetium. This observation aligns with findings by [56], which demonstrated that Fe-bound Tc(IV) can adopt an octahedral coordination and exhibit significant resistance to re-oxidation. Further investigation, particularly utilizing higher technetium concentrations to facilitate solid-phase analysis, is required to confirm and elucidate this incorporation phenomenon.

Evaluation of the engineered barrier system’s (ESB) degradation products revealed that bentonite clay significantly inhibited technetium immobilization, as evidenced by substantially reduced distribution coefficients. This attenuation is attributed to several mechanisms: bentonite can physically isolate corrosion products, thereby blocking access to their active sorption and redox sites [57] and creating a diffusion barrier that limits contact with pertechnetate ions [58]. Under the alkaline conditions expected in a repository, the use of compacted bentonite is likely to further suppress the pertechnetate reduction process by slowing the overall corrosion rate. Furthermore, Fe^2+^/Fe^3+^ cations released during corrosion may migrate into the interlayer spaces of smectite to compensate for structural charge deficits [59], effectively reducing the reactive surface area available for interaction with pertechnetate. The role of clay colloids in adsorbing and transporting iron species may also contribute significantly to this process [60,61].

It is noteworthy that certain iron-rich clay minerals like nontronite can facilitate the reductive immobilization of technetium via structural Fe(III) reduction, whether through chemical or microbial pathways [62]. Previous research has indicated that reduced iron—often associated with the activity of iron-reducing bacteria—whether sorbed on surfaces or within the crystal lattice, can promote technetium immobilization in various clays including montmorillonite, nontronite, and illite [63]. However, it is also established that corrosion products formed at the container-bentonite interface can adversely affect the clay’s chemical properties and buffering capacity [64,65].

In contrast to bentonite, the influence of cement and glass degradation products on technetium immobilization was less pronounced. The modest reduction in distribution coefficients (within 30%) observed with aluminophosphate glass leachates may be attributed to the formation of competing iron-phosphate phases that alter the sorptive surfaces [66]. The introduction of alkaline cement leachates, meanwhile, leads to solution alkalization and a consequent decrease in the steel corrosion rate, indirectly moderating the generation of reactive corrosion products [67].

## 5. Conclusions

The corrosion integrity of carbon steel containers is a fundamental factor governing the long-term stability of deep geological repository (DGR) systems. Predictive models indicate that the eventual ingress of low-salinity groundwater, characteristic of the prospective Yeniseysky DGR site, will induce areal corrosion of St3 steel, the designated material for vitrified radioactive waste containers. This scenario is anticipated to occur following the protracted degradation of primary engineered barriers, millennia after repository closure.

Given the 200,000-year half-life of technetium-99, accurate forecasting of its long-term environmental behavior is critical. Upon containment failure and contact with groundwater, technetium is likely to be released in the soluble form (TcO_4_^−^). However, our results demonstrate that Tc reductive immobilization onto iron corrosion products presents a significant retardation mechanism. Consequently, a carbon steel container can function not merely as a physical barrier, but also as an active geochemical barrier for technetium, a distinct advantage over stainless steel. It is important to note that using other materials for the container (stainless steel, copper) will not allow such a barrier to form. The efficacy of this process is strongly redox-dependent. Immobilization is most efficient under aerobic conditions, whereas under the anaerobic conditions expected to dominate a mature repository, significant technetium reduction is contingent upon the presence of specific, reactive corrosion products containing Fe(II), such as ferrihydrite. It is crucial to note that the presence of other engineered barrier materials, particularly bentonite clay, can diminish this immobilization efficiency by isolating reactive phases.

Within the planned multi-barrier system—comprising a steel canister, a clay buffer, and concrete seals—the most effective geochemical barrier for technetium is anticipated to be the interface between the steel canister and the clay. This zone is expected to exhibit distribution coefficients (*K_d_*) of 4–5 × 10^2^ cm^3^/g. Given the relatively low technetium inventory, these conditions favor the reduction and subsequent accumulation of the majority of the technetium. However, the gradual sorption of iron corrosion products onto the clay may deplete its reducing capacity. Consequently, if pertechnetate migrates into the clay buffer, its immobilization is predicted to decrease significantly, as the *K_d_* values for pertechnetate on bentonite are typically only a few cm^3^/g [68]. In a low-probability scenario involving buffer erosion due to high water flow, technetium could accumulate at the interface between steel corrosion products and cementitious materials. Furthermore, since steel is also used for tunnel reinforcement, technetium sorption onto their corroded surfaces is possible. Ultimately, the transport of corrosion products into the fractured rock mass, following the degradation of the engineered barriers, may lead to technetium immobilization on fracture surfaces.

This study primarily focuses on the deep geological disposal concept for radioactive waste, where oxidizing conditions are anticipated only during the initial operational phase prior to repository closure. Consequently, following closure, steel corrosion is expected to occur predominantly under anaerobic conditions, making the re-oxidation of technetium highly unlikely. However, the data obtained can also be applied to model technetium migration in near-surface, temporary storage facilities for technetium-bearing wastes. In such scenarios, steel tanks are subject to corrosion under aerobic conditions over time.

Since a definitive repository concept has not yet been finalized, this study utilized geochemical conditions and materials representative of potential conceptual designs. The current data do not allow for an evaluation of the role of bentonite clay type and compaction density on technetium migration in an engineered barrier system. Consequently, the findings presented here provide a foundational dataset that will be refined upon the selection of a specific repository concept.

Furthermore, predictive models for technetium migration must account for the complex interfacial interactions between the steel and bentonite. The corrosion-induced modification of the bentonite layer itself could, in theory, enhance its technetium retention properties. Indeed, the strategic modification of clay materials with iron-bearing compounds represents a promising avenue for developing advanced barrier systems tailored for the sequestration of long-lived radionuclides like technetium.

## Figures and Tables

**Table 1:** Elemental composition of corrosion products after 1 week (wt.%).

Point	O	S	Ca	Mn	Fe	Tc
1	23.01	0.69			68.57	7.73
2	23.42	1.00	0.16		58.05	17.37
3	26.73				28.08	45.19
4	22.41				64.50	13.09
5	22.34			0.39	70.66	6.61

**Table 2:** Values of the degree of immobilization and distribution coefficients (*K_d_*) of individual model corrosion products (A_0_ = 300 Bq/mL, S:L = 1:20).

Conditions	Aerobic Conditions		Anaerobic Conditions	
Sample	Degree of Immobilization, %	*K_d_*, cm^3^/g	Degree of Immobilization, %	*K_d_*, cm^3^/g
Corrosion product powder	96.6	568	34.2	134.8
FeO	98.9	1798	97.8	889
Ferrihydrite (Fe^3+^_10_O_14_(OH)_2_), freshly precipitated	98.8	1646	98.6	1409
Magnetite (Fe_3_O_4_)	3.1	0.6	0	0
Goethite (FeO(OH))	4.0	0.8	0	0
Hematite (Fe_2_O_3_)	5.1	1.1	0	0

**Table 3:** Efficiency of Tc removal from solution and values of interphase distribution coefficients (*K_d_*) during the interaction of corrosion product powder in solutions (Sample MW—corrosion product powder in model water with Tc; Sample MWG—addition aluminophosphate glass leachate; Sample MWC—cement leachate; Sample MWB—addition bentonite clay).

Sample	A/A_0_, %					*K_d_*, cm^3^/g
	0	1 h	4 h	24 h	168 h	
MW	100	58.1	27.8	3.4	3.4	568
MWG	100	57.3	28.5	6.9	6.8	474
MWC	100	56.6	27.3	4.8	4.7	406
MWB	100	65.9	33.8	13.3	13.0	134

## Data Availability

The original contributions presented in this study are included in the article/Supplementary Material. Further inquiries can be directed to the corresponding author.

## Supplementary Materials

The following supporting information can be downloaded at: https://www.mdpi.com/article/10.3390/ma18225220/s1, Table S1: Efficiency of pertechnetate ion removal from solution; Table S2: Elemental composition of corrosion products (at.%), Figure S1: Phase composition: (A) goethite, (B) hematite, (C) magnetite.

## Abbreviations

The following abbreviations are used in this manuscript:

ZVI	Zero-valent iron
DGR	Deep geological repository
XPS	X-ray photoelectron spectroscopy
SEM	Scanning electron microscopy
Eh	Oxidation Reduction Potential
S:L	Solid-to-liquid
