# Supplementary material for: Technetium Immobilization on Carbon Steel Corrosion Products Under Simulated Geological Radioactive Waste Repository Conditions
Source: Materials (Basel). 2025 Nov 18;18(22):5220. doi: 10.3390/ma18225220 (PMC12654470; doi:10.3390/ma18225220)
Supplement: Supplementary file 1 [file materials-18-05220-s001.zip › materials-3935334-supplementary.pdf]

Supplementary Materials

A

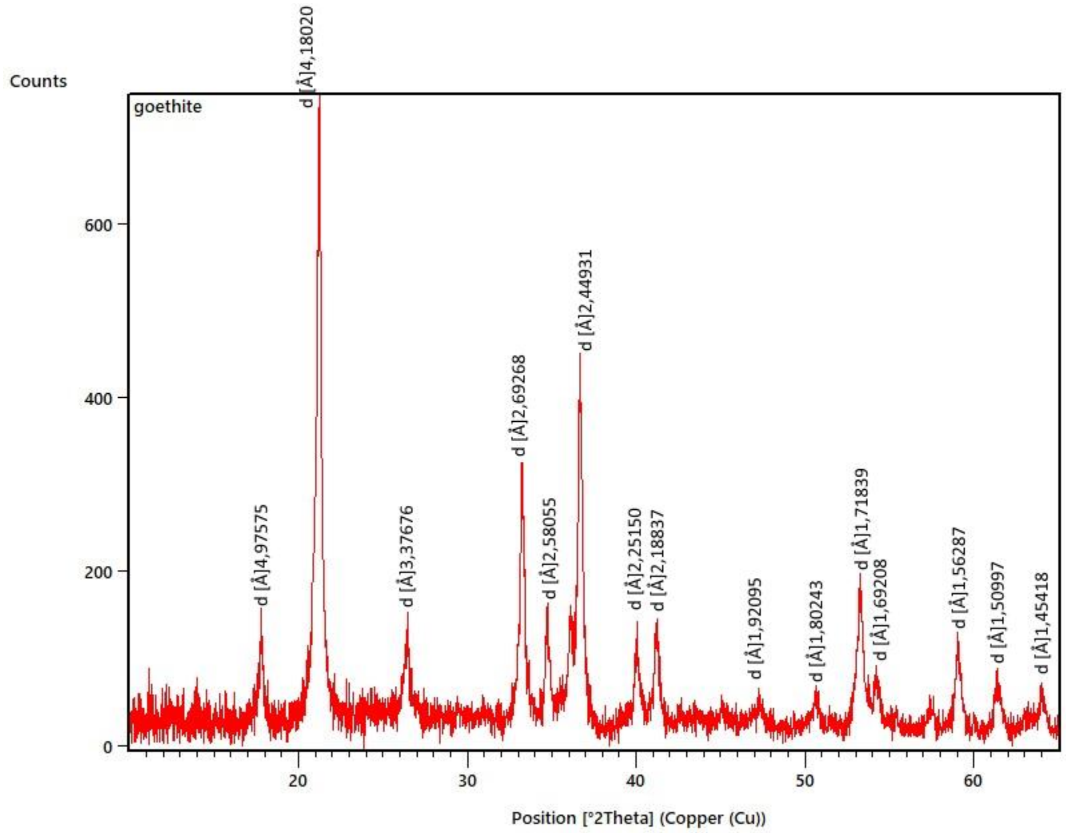

B

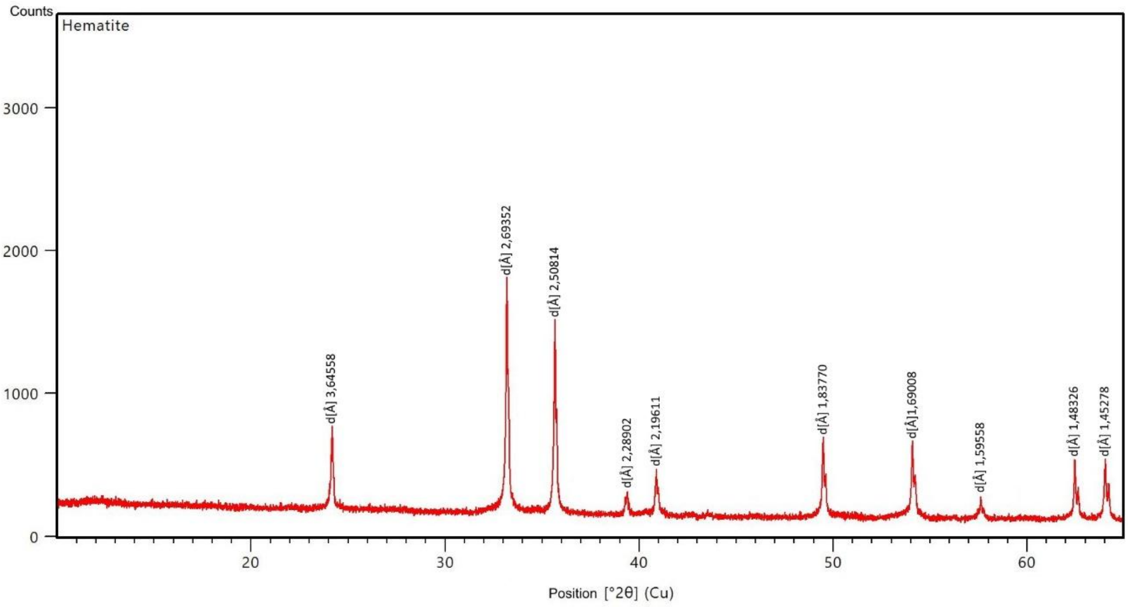

C

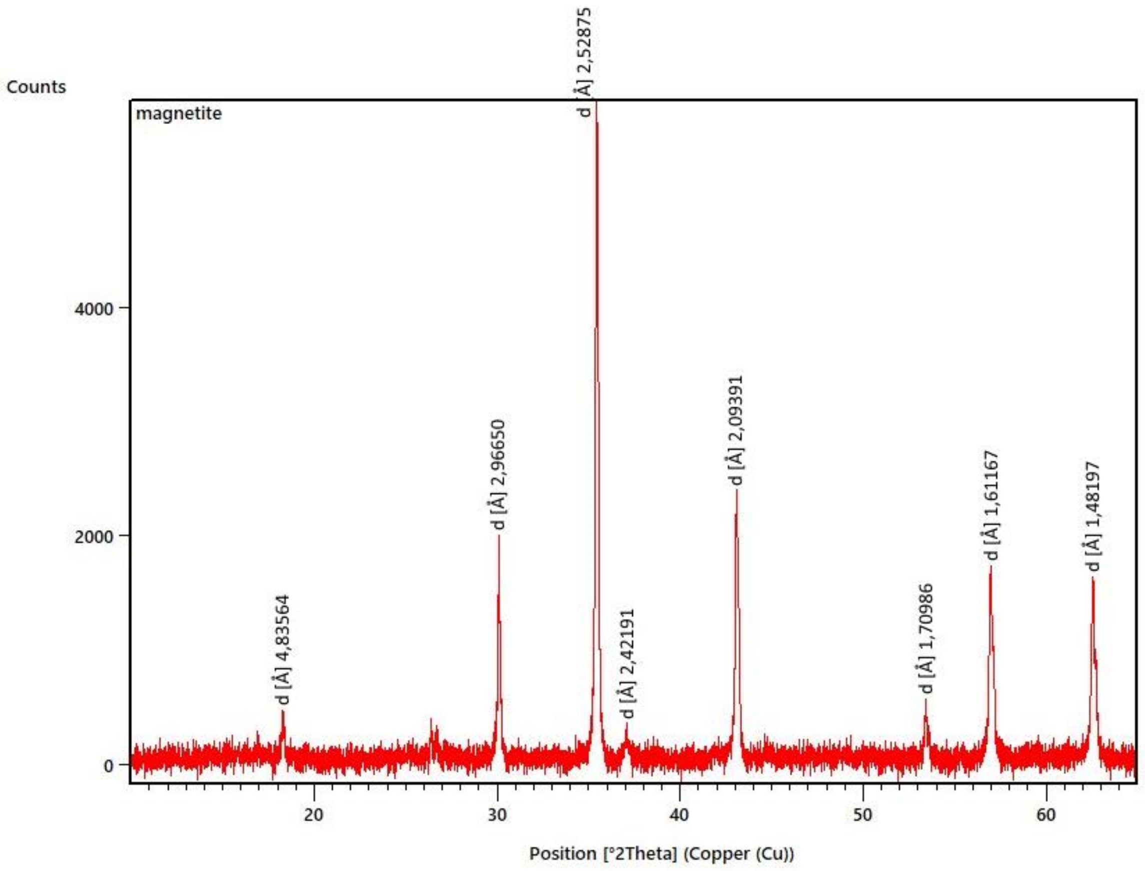

Figure S1. Phase composition: A) goethite, B) hematite, C) magnetite.

Table S1. Efficiency of pertechnetate ion removal from solution (%)

Sample	0h	1h	24h	168h
MW + Tc(VII) (without steel plate)	0	0	0	0
MW + Tc(VII) + steel with ingibitor IFKhAN-29 (aerobic conditions)	0	0	0	0
MW + Tc(VII) + steel with ingibitor IFKhAN-29 (anaerobic conditions)	0	0	0	0

Table S2. Elemental composition of corrosion products (at.%).

Point	**O**	**S**	**Ca**	**Mn**	**Fe**
			Figure 3		
1	52.13	0.19	0.13		47.55
2	51.58	0.17	0.11		48.14
3	52.15	0.51	0.16	0.14	47.04
4	52.48	1.1	0.14	0.14	46.14
			Figure 4		
5	50.21	0.21			49.58
6	50.23	0.18			49.49
			Figure 5		

7	51.9	0.13	0.09		47.88
8	52.1	0.27	0.12		47.51
9	51.74		0.13		48.13
10	51.38		0.12		48.5
